# Cannabinoids-Induced Rhabdomyolysis in a Patient: A Report of a Case and a Review of Literature

**DOI:** 10.7759/cureus.80809

**Published:** 2025-03-19

**Authors:** Sultan AlKhateeb, Bashaer Albulushi, Khaled Joueidi, Faisal Joueidi

**Affiliations:** 1 Department of Urology, King Faisal Specialist Hospital and Research Centre, Riyadh, SAU; 2 College of Medicine, Alfaisal University, Riyadh, SAU

**Keywords:** case report, drug induced, muscle injury, rhabdomyolysis, synthetic cannabinoids

## Abstract

Rhabdomyolysis is a life-threatening complication and is sometimes, though rarely, induced by the use of synthetic cannabinoids. The pathophysiology is poorly understood. However, the effect is through the activation of G-protein coupled receptors. We discuss the case of a 48-year-old man who was a known case of muscle-invasive bladder cancer and received four cycles of neoadjuvant chemotherapy. An X-ray showed an interval decrease in urinary bladder wall size and pelvic lymph nodes. He underwent radical cystoprostatectomy with lymph node dissection and an orthotopic new bladder. Postoperatively, the patient developed severe back pain, reduced lower limb function, and shortness of breath. Laboratory investigations revealed elevated creatinine and creatine kinase levels and decreased urine output. The diagnosis of rhabdomyolysis was made. Accordingly, the patient required continuous renal replacement therapy and was then shifted to hemodialysis. There are limited data available mentioning the effect of synthetic cannabinoids in the development of rhabdomyolysis. Our study emphasizes the importance of understanding the effects of synthetic cannabinoid toxicity in the development of rhabdomyolysis as well as of educating physicians and pharmacists in establishing better therapeutic and protective measures for satisfactory outcomes and minimizing the risk of mortality if left untreated.

## Introduction

Synthetic cannabinoids are heterogeneous groups of chemical substances that imitate the effect of marijuana and are potent agonists that bind to cannabinoid receptors 1 and 2 via the G-protein coupled receptors of the endocannabinoid system [1-3]. Cannabis is the most commonly abused illicit drug with an annual prevalence rate of 147 million individuals, accounting for 2.5% of the global population [4]. Rhabdomyolysis is a life-threatening condition that is caused by the excessive release of myoglobin into the circulation, resulting in the death of muscle tissues. Furthermore, it is considered a rare complication induced by the use of synthetic cannabinoids [5,6]. The pathophysiology of cannabinoids-induced rhabdomyolysis is poorly understood [5]. Thus, further studies are needed to determine the exact cause of such cases [1]. Synthetic cannabinoids have several effects on all biological systems, which have been described over the last few decades and have significantly impacted both behavioral and physiological systems. These include sensations of euphoria, relaxation, changes in memory and learning, loss of concentration, cognitive changes, psychosis, and schizophrenia [4]. Other physiological factors include dry mouth, increased appetite, and decreased respiratory rate [4]. The growing popularity of the use of synthetic cannabinoids for both therapeutic and recreational purposes, with limited scientific studies that discuss their toxicity and liability of abuse makes it challenging to appreciate their harmful and beneficial effects. Due to the wide variability in clinical manifestations, establishing laboratory investigations and a high index of suspicion is essential for early diagnosis and treatment [4-6]. The main management for cannabinoids-induced rhabdomyolysis includes supportive care followed by hemodialysis [5]. We recently treated a 48-year-old male who was diagnosed with rhabdomyolysis postoperatively and is on synthetic THC cannabinoids. This study aims to share our experience in linking the use of synthetic cannabinoid toxicity to the development of rhabdomyolysis and to establish better management options for those patients.

## Case presentation

A 48-year-old gentleman, who was medically on synthetic cannabis and was known to have muscle-invasive bladder cancer received neoadjuvant four cycles of cisplatin-gemcitabine chemotherapy. Post-chemotherapy images showed an interval decrease in the thickening of the urinary and the size of pelvic lymph nodes, Accordingly, the patient underwent radical cystoprostatectomy and lymph node dissection with orthotopic new bladder creation on November 26, 2023.

Operative details

The patient was placed in the supine position, anesthesia medication was administered intraoperatively (midazolam, propofol, fentanyl, rocuronium, cefazolin, metronidazole, heparin, calcium gluconate, furosemide, insulin, dexamethasone, sodium bicarb, and glycopyrrolate). The patient received a total of 5.5L IV fluids, had a 20 Fr Foley catheter inserted, had an estimated blood loss of 500 ml, and did not require inotropic support.

During postoperative care the following day, it was noticed that the patient was taking THC cannabinoids postoperatively as an incidental finding and had developed myoglobinuria, shortness of breath, severe back pain, and reduced lower limb function. Laboratory investigations showed transaminitis with increased aminotransferase (ALT), aspartate aminotransferase (AST), creatinine kinase (CK), and creatinine levels with reduced urine output (Table 1).

**Table 1:** Summary of postoperative laboratory values following cannabinoid intoxication

Laboratory workup	Results	Reference range
Hemoglobin (g/dL)	12 g/dL	>11.0 - <16.0 g/dL
Hematocrit (%)	38%	>32 - <47%
Myoglobulin	77 ng/mL	25 - 72 ng/mL
Serum Creatinine Kinase (CK) (U/L)	22,000 u/L	55 - 170 u/L
Creatinine	143 umol/L	64 - 115 umol/L
Urine Output (ml)	150 ml	800 - 2000 ml
Estimated Glomerular Filtration Rate (eGFR)	46 ml/min/1.73 m*2	>90 ml/min/1.73 m*2
Alanine Aminotransferase (ALT) (U/L)	65 u/L	7 - 55 u/L
Aspartate Aminotransferase (AST) (U/L)	54 u/L	8 - 48 u/L
Alkaline Phosphatase	47 IU/L	50 - 116 IU/L
Na	144 mEq/L	135 - 145 mEq/L
K	5.6 mmol/L	3.5 - 5.2 mmol/L
Ca	3.4 mmol/L	2.1 - 2.6 mmol/L
Mg	0.73 mg/dL	0.7 - 1 mg/dL
Phosphate	1.65 mmol/L	0.7 -1.4 mmol/L

The diagnoses of synthetic cannabinoid toxicity and rhabdomyolysis were made. The patient was overloaded and oliguric, where he initially required continuous renal replacement therapy (CRRT), then was shifted to hemodialysis and continued to receive continuous renal replacement therapy (CRRT) for a total of two days, and then machine clotted. After treatment, the patient’s edema and renal function started to gradually improve, and he began producing an adequate amount of urine and had a -ve balance. Additionally, the patient received two more sessions of hemodialysis. Afterward, his creatinine and creatinine kinase (CK) levels significantly improved, and he was producing adequate urine output (Table 2).

**Table 2:** Summary of laboratory values after treatment

Laboratory workup	Results	Reference range
Hemoglobin (g/dL)	14 g/dL	>11.0 - <16.0 g/dL
Hematocrits (%)	44%	>32 - <47 %
Myoglobulin	67 ng/mL	25 - 72 ng/mL
Serum Creatinine Kinase (CK) (U/L)	160 U/L	55 - 170 U/L
Creatinine	87 umol/L	52.2 - 91.9 umol/L
Urine Output (ml)	900 ml	800 - 2000 ml
Estimated Glomerular Filtration Rate (eGFR)	78 ml/min/1.73 m*2	>90 ml/min/1.73 m*2
Alanine Aminotransferase (ALT) (U/L)	52 U/L	7 - 55 u/L
Aspartate Aminotransferase (AST) (U/L)	41 U/L	8 - 48 u/L
Alkaline Phosphatase	46 IU/L	50 - 116 IU/L
Na	135 mEq/L	135 - 145 mEq/L
K	4.8 mmol/L	3.5 – 5.2 mmol/L
Ca	2.3 mmol/L	2.1 - 2.6 mmol/L
Mg	0.02 mg/dL	0.7 - 1 mg/dL
Phosphate	1.3 mmol/L	0.7 - 1.4 mmol/L

Nephrology followed the patient throughout his admission. His creatinine, creatinine kinase, and myoglobulin values returned to normal levels. He was fit for discharge and for follow-up in the local hospital for his chronic kidney disease (CKD). On follow-up in the urology clinic (January 1, 2024), his cystogram showed no evidence of a leak. He was counseled about clean intermittent catheterization (CIC) and bladder irrigation and was scheduled to follow up three months later.

## Discussion

Synthetic cannabinoids are heterogeneous biological compounds that act in binding to cannabinoid receptors of psychoactive substrates with a high potency [7,8]. Endocannabinoids are a compound modularity system that is responsible for cell response to various intrinsic and extrinsic stimulants through a complex cascade of gene expression, receptor activation, and enzyme reactions [8]. Exposure to cannabinoids in either synthetic or organic form is associated with physical and psychological factors [8]. Rhabdomyolysis is a disorder that is characterized by muscle injury resulting in the leakage of intracellular myocytes into the extracellular space [9]. The etiology is associated with several factors, including trauma, infection, muscle ischemia, dehydration, exercise, heat stroke, and drug intoxication (cannabinoids, heroin, and cocaine) [7,10]. Drug-induced rhabdomyolysis is divided into primary and secondary myotoxic effects [10]. Rhabdomyolysis usually presents with a wide variety of clinical features ranging from muscle weakness to life-threatening acute renal failure [3]. The classical triad includes skeletal muscle injury, dark urine, and renal dysfunction [10]. However, in drug-induced rhabdomyolysis, subclinical features can present without common features [10]. Studies showed that drugs are the most frequent causative agents, reaching 81% of cases [11].

The pathophysiology of cannabinoids-induced rhabdomyolysis is poorly understood [1]. However, studies suggest that the effect of synthetic cannabinoids is carried out through two receptors, CB1 and CB2. The CB1 receptors are considered the responsive form of the cannabinoid component that activates G-protein coupled receptors in the presynaptic terminal, decreasing the cellular cyclic adenosine monophosphate (cAMP) levels and eliciting a cannabimimetic response [12]. CB2 mainly controls the release of cytokine and the migration of immune cells [12]. However, the binding affinity is greater in CB1 in comparison to CB2 [13]. The impairment in the production of endocannabinoids can dysregulate the CB1 receptor and their signaling pathways, contributing to the irreversible degeneration of skeletal muscle tissue [8]. Another mechanism is the dysregulation of Krebs cycle activation in the mitochondrial membrane, altering the glucose and fatty acid metabolism and affecting the skeletal muscle fibers [8]. Studies also showed that metabolites such as the inhibition of gamma-aminobutyric acid can relatively increase creatinine kinase (CK) levels and result in kidney failure, which can subsequently manifest in the development of rhabdomyolysis [14]. Ronco et al. showed that conventional dialysis techniques can limit the capacity of removing myoglobin from circulation because of molecular properties, solute transport mechanisms, or extracorporeal technical difficulties [14]. Amanollahi et al. reported a study in Iran showed that among 227 poisoned individuals, almost 75.8% of those affected were men. The reason behind this is that women have a higher mitochondrial mass in the skeletal muscle with a greater oxidative phosphorylative capacity, and therefore, greater protection against rhabdomyolysis [7].

Cannabinoids-induced rhabdomyolysis is a diagnosis of exclusion. Furthermore, establishing a high index of suspicion is essential for early identification through a detailed history, clinical evaluation, and laboratory investigations, including negative universal drug screens [1,6]. The management of such cases includes continuous elimination of the exposed drug, infusion of intravenous normal saline early, sufficient volume replenishment, and electrolyte correction. The goal is to cease muscle destruction, resolve metabolic and electrolyte abnormalities, and preserve renal function [5,10]. Paul et al. reported two cases who presented with nausea, vomiting, and generalized fatigue and had elevated CK of 47,000 and 37,000 U/L, respectively. The diagnosis of rhabdomyolysis was made. Accordingly, the patients underwent aggressive hydration and forced diuretics followed by hemodialysis [1]. Strain et al. reported a 26-year-old who presented with weakness and severe muscle pain after exercise, which was suggestive of rhabdomyolysis. Accordingly, the patient underwent aggressive hydration with normal saline [5]. Another study by Adedinsewo et al. reported on patients who ingested cannabinoids and presented with seizures, agitation, and bizarre behavior (Table 3) [6]. In our case, the patient had increased serum CK levels with decreased urine output postoperatively, and it was discovered that he was administering synthetic THC cannabinoids and was intoxicated, which led to the development of rhabdomyolysis. Accordingly, he received aggressive hydration therapy with normal saline and underwent continuous renal replacement therapy (CRRT) and then was shifted to hemodialysis where his creatinine kinase, myoglobin creatinine, and urine output gradually improved.

**Table 3:** Summary of all reported cases in the literature, outlining the demographics, presenting symptoms, and treatment options

Article	Age	Gender	Symptoms	Treatment
Our Case	48-year-old	Male	Hematuria, shortness of breath, severe back pain with reduced lower limb function	Renal replacement therapy and then was shifted to hemodialysis
Strain et al. (2019) [2]	26-year-old	Male	Weakness and severe muscle pain	Hydration with normal saline
Paul et al (2016) [1]	Case 1: 30-year-old; Case 2: 23-year-old	Case 1: Male; Case 2: Male	Case 1: Nausea, vomiting, abdominal pain, and difficulty urinating; Case 2: nausea, vomiting, chest pain, generalized weakness, body aches, and muscle cramps	Case 1: Hydration and forced diuretics followed by hemodialysis; Case 2: Hydration and forced diuretics followed by hemodialysis
Adedinsewo et al. (2016) [3]	-	Male	Seizure, agitation, and bizarre behavior	Hydration therapy with normal saline

## Conclusions

Synthetic cannabinoid-induced rhabdomyolysis is a rare, life-threatening complication that should be addressed in medical practice. Despite the growing concern about the increased rates of illegal cannabinoid usage in society, there is limited data available outlining the effect of synthetic cannabinoids in the development of rhabdomyolysis. It is important to educate physicians and pharmacists about the effect of synthetic cannabinoid toxicity by providing the appropriate supportive care, managing symptoms for better outcomes, and preventing mortality among affected individuals.
